# Inherited pathogenic mitochondrial DNA mutations and gastrointestinal stem cell populations

**DOI:** 10.1002/path.5156

**Published:** 2018-11-05

**Authors:** Tianhong Su, John P Grady, Sorena Afshar, Stuart AC McDonald, Robert W Taylor, Doug M Turnbull, Laura C Greaves

**Affiliations:** ^1^ Wellcome Centre for Mitochondrial Research Institute of Neuroscience, Newcastle University Newcastle upon Tyne UK; ^2^ Human Nutrition Research Centre, Institute of Cellular Medicine, Newcastle University, Campus for Ageing and Vitality Newcastle upon Tyne UK; ^3^ Centre for Tumour Biology, Barts Cancer Institute, Queen Mary University of London London UK; ^4^ LLHW Centre for Ageing and Vitality, Newcastle University Institute for Ageing, The Medical School Newcastle upon Tyne UK

**Keywords:** mitochondrial DNA mutation, selection, segregation, mitochondrial disease, intestinal stem cell, gastrointestinal epithelium, MELAS, MERRF, m.3243A>G, alimentary canal

## Abstract

Inherited mitochondrial DNA (mtDNA) mutations cause mitochondrial disease, but mtDNA mutations also occur somatically and accumulate during ageing. Studies have shown that the mutation load of some inherited mtDNA mutations decreases over time in blood, suggesting selection against the mutation. However, it is unknown whether such selection occurs in other mitotic tissues, and where it occurs within the tissue. Gastrointestinal epithelium is a canonical mitotic tissue rapidly renewed by stem cells. Intestinal crypts (epithelium) undergo monoclonal conversion with a single stem cell taking over the niche and producing progeny. We show: (1) that there is a significantly lower mtDNA mutation load in the mitotic epithelium of the gastrointestinal tract when compared to the smooth muscle in the same tissue in patients with the pathogenic m.3243A>G and m.8344A>G mutations; (2) that there is considerable variation seen in individual crypts, suggesting changes in the stem cell population; (3) that this lower mutation load is reflected in the absence of a defect in oxidative phosphorylation in the epithelium. This suggests that there is selection against inherited mtDNA mutations in the gastrointestinal stem cells that is in marked contrast to the somatic mtDNA mutations that accumulate with age in epithelial stem cells leading to a biochemical defect. © 2018 The Authors. *The Journal of Pathology* published by John Wiley & Sons Ltd on behalf of Pathological Society of Great Britain and Ireland.

## Introduction

Mitochondria are ubiquitous organelles present in eukaryotic cells, a major function of which is to generate ATP via the process of oxidative phosphorylation (OXPHOS). Mitochondria contain their own DNA (mtDNA), which encodes 13 essential protein subunits of the OXPHOS system, 22 tRNAs and 2 rRNAs. Each cell contains multiple copies of mtDNA. Cells may be homoplasmic, where all the copies of mitochondrial DNA are identical, or heteroplasmic, with a mixture of mutated and wild‐type mtDNA molecules. The vast majority of mtDNA mutations are functionally recessive and an OXPHOS defect only occurs when the mutation load exceeds a critical threshold 1.

Mutations of mtDNA are a common cause of human inherited disease 2, but they also accumulate somatically in tissues such as skeletal muscle, intestinal epithelium, and blood with age 3, 4, 5. In patients with primary heteroplasmic mtDNA disease, despite uniform mutation load across all tissues during foetal development 6, 7, 8, some mtDNA mutations show a decrease in the mutation load with age in blood and a few epithelial tissues 9, 10, 11. This suggests that mitotic tissue may selectively lose inherited mtDNA mutations over time. This is in marked contrast to somatic mtDNA mutations that accumulate in such tissues with age 4, 5, 12. In addition, it is unknown whether the loss of inherited mtDNA mutations is a common feature for all mitotic tissues and where it happens in the tissue. To address some of these questions, we have investigated germline pathogenic heteroplasmic mtDNA mutations in gastrointestinal stem cell populations. We compared OXPHOS activity, mitochondrial protein expression, and mutation load in the epithelium and smooth muscle of the oesophagus, stomach, and the small and large intestines of patients with the common inherited m.3243A>G mtDNA variant within MT‐TL1 (encoding mitochondrial tRNA^Leu(UUR)^) and m.8344A>G mtDNA variant within MT‐TK (encoding mitochondrial tRNA^Lys^).

## Materials and methods

### Patients

Gastrointestinal tissue samples were collected from three patients with m.3243A>G (patient 1 following ileum resection at the age of 30; patients 2 and 3, aged 36 and 64, respectively at post‐mortem) and from one patient with m.8344A>G aged 56. Control tissue was either taken during endoscopy from patients in whom no pathology was found or at resection for colon cancer at a distance of > 20 cm from the neoplasm. Details of the subjects may be found in the supplementary material, Supplementary materials and methods and Tables S1 and S2. Ethical approval was obtained from Newcastle and North Tyneside LREC.

### Pyrosequencing

**Figure 1:**
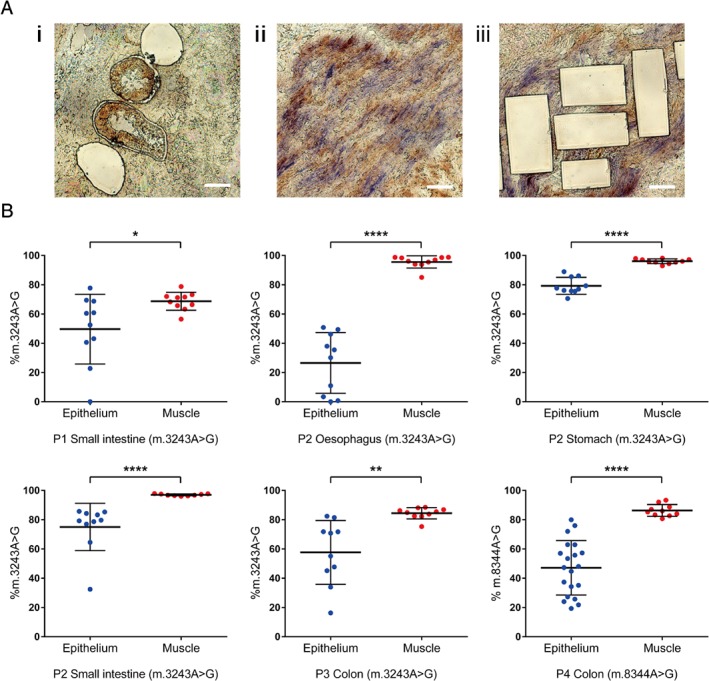
Lower levels of m.3243A>G and m.8344A>G detected in the mitotic epithelium compared with the post‐mitotic smooth muscle of GI tract tissues. (A) Representative images of laser‐microdissected (i) intestinal crypts and smooth muscles before (ii) and after (iii) laser microdissection. (B) Quantitative pyrosequencing showing the mutation levels of inherited m.3243A>G in the epithelium and the smooth muscle of the oesophagus, the stomach, the small intestine (SI), and the colon from three patients, and inherited m.8344A>G in the colonic epithelium and smooth muscle of one patient. Each replicate represents DNA extracted from five pooled crypts of the intestines, five gastric pits of the stomach or five small areas of smooth muscle. Oesophageal DNA was obtained from the tissue laser cut as an intact area in a field of view. P1 SI (*n*_e_ = 10, *n*_m_ = 10), P2 oesophagus (*n*_e_ = 10, *n*_m_ = 10), P2 stomach (*n*_e_ = 10, *n*_m_ = 10), P2 SI (*n*_e_ = 10, *n*_m_ = 9), P3 colon (*n*_e_ = 10, *n*_m_ = 10), P4 colon (*n*_e_ = 20, *n*_m_ = 10). *p < 0.05, **p < 0.005, and ****p < 0.0001 by unpaired *t*‐test or Mann–Whitney *U*‐test based on the normality of the data.

Epithelial crypts and smooth muscle fibres were randomly selected and laser‐microdissected using a PALM Laser microdissection system (Zeiss, Jena, Germany) (Figure 1A). Total DNA was extracted by cell lysis as previously described 13. PyroMark Assay design software v2.0 (QIAGEN, Hilden, Germany) was used to design the primer trio for pyrosequencing (supplementary material, Table S3). Heteroplasmy levels were quantified using the PyroMark Q96 software according to the manufacturer's instructions.

### Sequential Cytochrome *c* Oxidase/Succinate Dehydrogenase (COX/SDH) histochemistry

Sequential COX/SDH histochemistry was carried out as previously described 4. Quantification of COX deficiency was calculated as the proportion of COX‐deficient crypts by all the crypts counted on two sections.

### Immunofluorescence

Quadruple immunofluorescence was performed as previously described 14. Details of the antibodies used may be found in the supplementary material, Table S4. The optical density of the fluorescent images was measured by ImageJ. Background correction and the method to determine the parameters (mean and standard deviation, SD) of the control population have been described formerly 14. The *Z*‐score of each respiratory chain subunit for the disease case was calculated and categorised based on the normal population. For NDUFB8, the level was classified as ‘negative’ (< −3 SD), ‘intermediate’ (−3 to −2 SD) or ‘positive’ (> −2 SD). For the non‐mtDNA encoded proteins COX4 and SDHA, the levels were categorised as ‘low’ (< −2 SD), ‘normal’ (−2 to 2 SD) or ‘high’ (> 2 SD).

## Results

### MtDNA mutation load in epithelial crypts compared with smooth muscle fibres

The load of the m.3243A>G mutation was lower in crypts laser‐microdissected from the mucosa compared with fibres from the smooth muscle in oesophagus (*p* < 0.0001, Mann–Whitney *U*‐test), stomach (*p* < 0.0001, unpaired *t*‐test), the small intestines (SI) (*p* < 0.05 for patient 1 and *p* < 0.0001 for patient 2, unpaired *t*‐test and Mann–Whitney *U*‐test respectively), and colon (*p* < 0.005, unpaired *t*‐test) (Figure 1B). In addition, our studies showed a markedly lower heteroplasmy in the mitotic epithelium than in the smooth muscle of colon from the patient with the m.8344A>G mutation (*p* < 0.0001, unpaired *t*‐test) (Figure 1B). The mutation load in the epithelium was notably variable, with no detectable mutation in some of the intestinal crypts and oesophageal epithelium. We did not observe any crypts with m.3243A>G higher than 86% or any oesophageal epithelium with m.3243A>G higher than 51%. The colonic epithelium also showed an upper threshold for m.8344A>G of approximately 80%. In most cases, the level in the mucosal epithelium was also lower than other post‐mitotic tissues such as skeletal and cardiac muscle, suggesting a loss of mutation overall (supplementary material, Table S2).

### Enzyme activity and protein level of OXPHOS complexes

**Figure 2:**
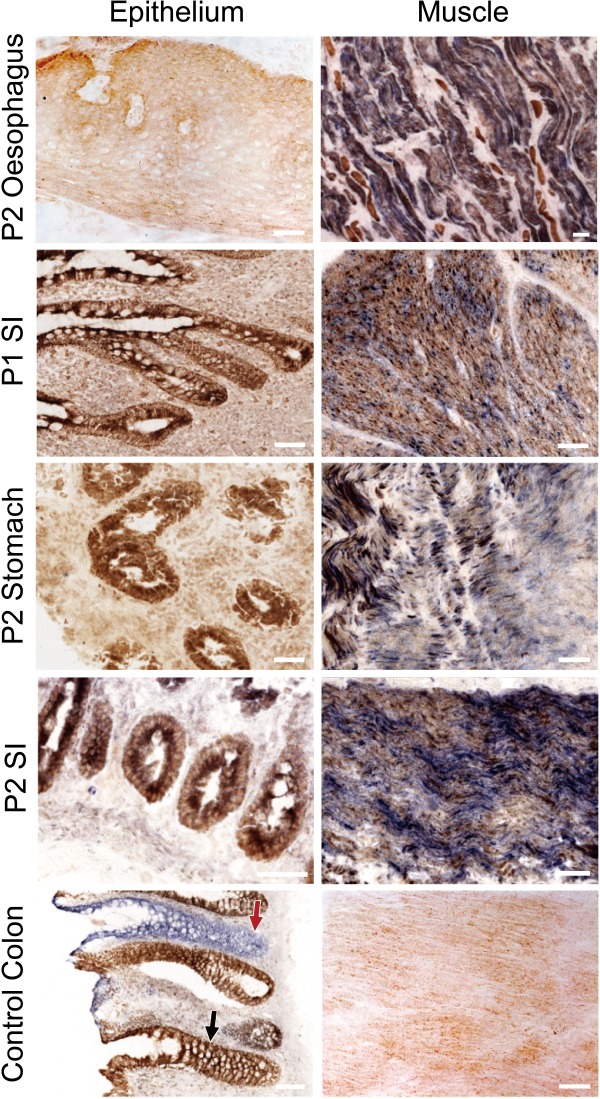
Deficient COX activity in post‐mitotic smooth muscle but normal COX activity in mitotic epithelium of the alimentary canal in patients with inherited m.3243A>G. The left panel shows the COX‐normal epithelium that is labelled brown in the SI of patient 1, and the oesophagus, the stomach, and the SI of patient 2, while the right panel manifests the blue COX‐deficient muscle fibres in these tissues. The control panel shows the COX‐normal epithelium (black arrow) and smooth muscle from a normal individual who also has crypts with defective COX activity (red arrow), due to accumulated somatic mtDNA mutations during ageing. Scale bar = 50 μm.

The m.3243A>G mutation in the tRNA^Leu(UUR)^ gene impairs mitochondrial protein synthesis, causing defects in single or multiple respiratory chain complexes, including complex I and complex IV (COX) enzyme activity 15. We hypothesised that if the inherited pathogenic mutation was lost in the epithelium, this would alleviate the mitochondrial biochemical defect within the tissue 15. Sequential COX/SDH histochemistry showed very little COX activity in the post‐mitotic smooth muscle of all three regions of the gastrointestinal tract. However, COX activity was largely preserved in the epithelial cells (Figure 2). We detected no COX deficiency in the oesophagus and SI of patient 2, and only 1.25% COX deficiency in the SI epithelium of patient 1, 2.67% in the stomach epithelium of patient 2, and 2.59% in the large intestinal epithelium of patient 3.

**Figure 3:**
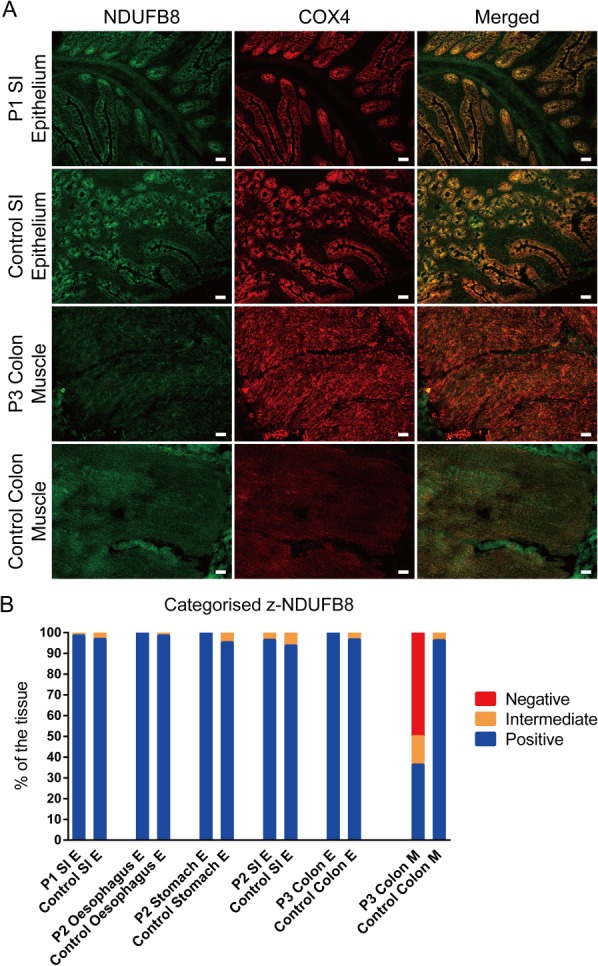
Protein levels of complex I were normal in the mitotic epithelium along the gastrointestinal tract but deficient in the post‐mitotic smooth muscles. (A) Example of immunofluorescence images showing the levels of NDUFB8 (complex I) (green) and COX4 (red), a nuclear encoded subunit that is not compromised by the m.3243A>G mutation. (B) Quantative measurement of the protein level of NDUFB8. *Z*‐scores of NDUFB8 for each patient were calculated and categorised based on the age‐matched control population. The numbers of crypts for quantification were as follows: *n* (P1 SI) = 70; *n* (control) =128; *n* (P2 SI) = 28; *n* (control) = 48; *n* (P2 stomach) = 6; *n* (control) = 36; *n* (P3 colon) = 20; *n* (control) = 91. Oesophageal epithelium and colonic smooth muscle from the whole section were selected for quantification. Patient data were compared with data from two controls for the stomach; three controls for the colon, the oesophagus, and the SI of patient 2; and four controls for the SI of patient 1. E = epithelium. M = muscle. Scale bar = 50 μm

Since there is no reliable histochemical assay for complex I, we used immunofluorescence to quantify the levels of NDUFB8, a subunit of respiratory chain complex I critical to the complex assembly and often lost in OXPHOS deficiency 14. NDUFB8 showed normal expression in the epithelium of the colon, oesophagus, stomach, and SI of the patients (Figure 3) compared with age‐matched controls. In contrast, NDUFB8 was low with 50% deficiency in the post‐mitotic smooth muscle of the colon from patients compared with age‐matched controls (Figure 3). We found higher levels of nuclear encoded mitochondrial proteins, SDHA and COX4, both of which are reported to be preserved in tissues with mtDNA defects 16, in the epithelium of the patients' oesophagus (47.62% and 40.91%, respectively), stomach (14.29% for SDHA), and SI (10% in patient 2 for SDHA; 8.57% in patient 1 and 14.29% in patient 2 for COX4) (supplementary material, Figure S1). SDHA also increased in the colonic muscle of the patient (27.27% (supplementary material, Figure S1)). This is likely a compensatory response.

## Discussion

Understanding the behaviour of mtDNA mutations in different tissues is critical not only to understanding the phenotype of inherited mtDNA disease but also in our understanding of the impact of acquired mtDNA mutations seen in human ageing. Here, we have investigated multiple epithelial tissues from patients with inherited mtDNA mutations and have shown a significantly lower mtDNA mutation level in epithelial cells compared with the post‐mitotic smooth muscle fibres of the oesophagus, the stomach, and the small and the large intestine. We show that the mutation level is correlated with the finding of normal COX activity and complex I protein levels in epithelial cells, but deficient COX activity and low complex I protein expression in the post‐mitotic smooth muscle from the same patients. The finding of respiratory chain deficiency in the gastrointestinal smooth muscle is similar to previous reports 17 and entirely consistent with the severe symptoms of bowel dysmotility in many patients with mitochondrial disease.

Previous reports in foetal tissues show that the level of mtDNA mutation was largely uniform in all tissues 6, 7, 8. Given that there is little evidence that the mutation burden changes with age in muscle 10, our results suggest a loss of inherited mtDNA mutation in the mitotic gastrointestinal epithelium with age. This is consistent with previous reports showing a loss of the m.3243A>G mutation in patients' blood over time 9, 10. However, as all of our patients are adults, the exact time of the loss remains unknown. It is known that m.3243A>G mutation load is the same in all tissues during embryo development and fetal growth 6, 7, 8 and the studies in blood (where serial measurements are possible) show loss of mutation throughout life but most markedly in the early years 9, 10. Whilst we have a very small patient cohort, we did determine if there was a trend for more mutation loss in epithelial cells in the older patient (64 years) when compared with the same type of epithelial tissue from the younger patient (30 years). We did not detect a difference but previous studies in blood have shown considerable individual variation and a slowing down of selection after early adult life 10.

The site of the loss of mutation in mitotic tissues is unknown but previous *in silico* modelling suggests that the selective loss occurs in haematopoietic stem cells 18. Intestinal crypts have stem cells at the base with amplifying cells and differentiated progenies present in the crypt. Crypts also undergo monoclonal conversion until a whole crypt derives from a single stem cell 19. In this context, the pattern of mtDNA mutation load seen in crypts is interesting since it shows marked variation in the level, with some crypts carrying no detectable mutation. This strongly indicates that selection against the mutation is occurring at the stem cell level since selection at any other stage is unlikely to result in no detectable mutation. The overall marked decrease in the mutation level and the upper cut‐off threshold in the intestinal crypts imply a negative selection against the mutations, not a bidirectional random genetic drift.

These observations in intestinal crypts in patients with mtDNA disease are in marked contrast to the observation in normal ageing of human gastrointestinal stem cells, where mitochondrial DNA mutations accumulate somatically up to homoplasmy, resulting in OXPHOS deficiency 4, 12, 20. In contrast to our data for inherited mtDNA mutations, there is no evidence of any selective pressures on these somatic mtDNA mutations 21. The difference in the selective pressures on somatic and inherited mtDNA mutations remains unknown but indicates significant changes in stem cell biology in the normal ageing process.

## Author contributions statement

LCG and DMT conceived and designed the study. SA, SACM, and RWT provided study material. TS collected and/or assembled data. TS and JPG analysed and interpreted data. TS, LCG, and DMT wrote the manuscript. TS, JPG, SA, SACM, RWT, DMT, and LCG had final approval of the manuscript.

**SUPPLEMENTARY MATERIAL ONLINE**

**Supplementary materials and methods**

**Supplementary figure legends**

**Figure S1.** Quantitative measurement of COX4 and SDHA level in the gastrointestinal epithelium and smooth muscle

**Table S1.** Information of the subjects and the obtained tissue

**Table S2.** Heteroplasmic levels of pathogenic mtDNA mutations measured in various tissues of the four patients

**Table S3.** Primer sequences used for pyrosequencing to quantify m.3243A>G and m.8344A>G mutation levels

**Table S4.** Antibodies and concentrations used in the immunofluorescence assay

## Supporting information


**Supplementary materials and methods**

**Supplementary figure legends**

**Figure S1.** Quantitative measurement of COX4 and SDHA level in the gastrointestinal epithelium and smooth muscle

**Table S1.** Information of the subjects and the obtained tissue

**Table S2.** Heteroplasmic levels of pathogenic mtDNA mutations measured in various tissues of the four patients

**Table S3.** Primer sequences used for pyrosequencing to quantify m.3243A>G and m.8344A>G mutation levels

**Table S4.** Antibodies and concentrations used in the immunofluorescence assay

## References

[1] Taylor RW , Turnbull DM . Mitochondrial DNA mutations in human disease. Nat Rev Genet 2005; 6: 389–402. 15861210 10.1038/nrg1606 PMC1762815

[2] Gorman GS , Schaefer AM , Ng Y , *et al* Prevalence of nuclear and mitochondrial DNA mutations related to adult mitochondrial disease. Ann Neurol 2015; 77: 753–759. 25652200 10.1002/ana.24362 PMC4737121

[3] Bua E , Johnson J , Herbst A , *et al* Mitochondrial DNA‐deletion mutations accumulate intracellularly to detrimental levels in aged human skeletal muscle fibers. Am J Hum Genet 2006; 79: 469–480. 16909385 10.1086/507132 PMC1559550

[4] Taylor RW , Barron MJ , Borthwick GM , *et al* Mitochondrial DNA mutations in human colonic crypt stem cells. J Clin Invest 2003; 112: 1351–1360. 14597761 10.1172/JCI19435 PMC228466

[5] Shin MG , Kajigaya S , Tarnowka M , *et al* Mitochondrial DNA sequence heterogeneity in circulating normal human CD34 cells and granulocytes. Blood 2004; 103: 4466–4477. 15016645 10.1182/blood-2003-11-3949

[6] Matthews PM , Hopkin J , Brown RM , *et al* Comparison of the relative levels of the 3243 (A→G) mtDNA mutation in heteroplasmic adult and fetal tissues. J Med Genet 1994; 31: 41–44. 8151636 10.1136/jmg.31.1.41 PMC1049597

[7] Cardaioli E , Fabrizi GM , Grieco GS , *et al* Heteroplasmy of the A3243G transition of mitochondrial tRNA^Leu(UUR)^ in a MELAS case and in a 25‐week‐old miscarried fetus. J Neurol 2000; 247: 885–887. 11151425 10.1007/s004150070080

[8] Monnot S , Gigarel N , Samuels DC , *et al* Segregation of mtDNA throughout human embryofetal development: m. 3243A> G as a model system. Hum Mutat 2011; 32: 116–125. 21120938 10.1002/humu.21417 PMC3058134

[9] Rahman S , Poulton J , Marchington D , *et al* Decrease of 3243 A→ G mtDNA mutation from blood in MELAS syndrome: a longitudinal study. Am J Hum Genet 2001; 68: 238–240. 11085913 10.1086/316930 PMC1234919

[10] Grady JP , Pickett SJ , Ng YS , *et al* mtDNA heteroplasmy level and copy number indicate disease burden in m.3243A>G mitochondrial disease. EMBO Mol Med 2018; 10: e8262. 10.15252/emmm.201708262 PMC5991564 29735722

[11] Olsson C , Johnsen E , Nilsson M , *et al* The level of the mitochondrial mutation A3243G decreases upon ageing in epithelial cells from individuals with diabetes and deafness. Eur J Hum Genet 2001; 9: 917–921. 11840193 10.1038/sj.ejhg.5200742

[12] Greaves LC , Barron MJ , Plusa S , *et al* Defects in multiple complexes of the respiratory chain are present in ageing human colonic crypts. Exp Gerontol 2010; 45: 573–579. 20096767 10.1016/j.exger.2010.01.013 PMC2887930

[13] Rygiel KA , Grady JP , Taylor RW , *et al* Triplex real‐time PCR – an improved method to detect a wide spectrum of mitochondrial DNA deletions in single cells. Sci Rep 2015; 5: 9906. 25989140 10.1038/srep09906 PMC4437295

[14] Rocha MC , Grady JP , Grünewald A , *et al* A novel immunofluorescent assay to investigate oxidative phosphorylation deficiency in mitochondrial myopathy: understanding mechanisms and improving diagnosis. Sci Rep 2015; 5: 15037. 10.1038/srep15037 PMC4606788 26469001

[15] Hämäläinen RH , Manninen T , Koivumäki H , *et al* Tissue‐ and cell‐type‐specific manifestations of heteroplasmic mtDNA 3243A>G mutation in human induced pluripotent stem cell‐derived disease model. Proc Natl Acad Sci U S A 2013; 110: E3622–E3630. 24003133 10.1073/pnas.1311660110 PMC3780874

[16] Chrysostomou A , Grady JP , Laude A , *et al* Investigating complex I deficiency in Purkinje cells and synapses in patients with mitochondrial disease. Neuropathol Appl Neurobiol 2016; 42: 477–492. 26337858 10.1111/nan.12282 PMC4973693

[17] Betts J , Barron MJ , Needham SJ , *et al* Gastrointestinal tract involvement associated with the 3243A> G mitochondrial DNA mutation. Neurology 2008; 70: 1290–1292. 18391161 10.1212/01.wnl.0000308940.38092.74

[18] Rajasimha HK , Chinnery PF , Samuels DC . Selection against pathogenic mtDNA mutations in a stem cell population leads to the loss of the 3243A→G mutation in blood. Am J Hum Genet 2008; 82: 333–343. 18252214 10.1016/j.ajhg.2007.10.007 PMC2427290

[19] Snippert HJ , van der Flier LG , Sato T , *et al* Intestinal crypt homeostasis results from neutral competition between symmetrically dividing Lgr5 stem cells. Cell 2010; 143: 134–144. 20887898 10.1016/j.cell.2010.09.016

[20] McDonald SAC , Greaves LC , Gutierrez‐Gonzalez L , *et al* Mechanisms of field cancerization in the human stomach: the expansion and spread of mutated gastric stem cells. Gastroenterology 2008; 134: 500–510. 18242216 10.1053/j.gastro.2007.11.035

[21] Greaves LC , Elson JL , Nooteboom M , *et al* Comparison of mitochondrial mutation spectra in ageing human colonic epithelium and disease: absence of evidence for purifying selection in somatic mitochondrial DNA point mutations. PLoS Genet 2012; 8: e1003082. 10.1371/journal.pgen.1003082 PMC3499406 23166522

