# Supplementary material for: Inherited pathogenic mitochondrial DNA mutations and gastrointestinal stem cell populations
Source: J Pathol. 2018 Nov 5;246(4):427–32. doi: 10.1002/path.5156 (PMC6282723; doi:10.1002/path.5156)
Supplement: Supplementary file 1 — Supplementary materials and methods [file PATH-246-427-s001.docx]

**Inherited pathogenic mitochondrial DNA mutations and gastrointestinal stem cell populations**

**Su T *et al.* J Pathol 2018 (DOI: 10.1002/path.5156)**

### Supplementary materials and methods

### Case history

Patient 1, harbouring the m.3243A>G mutation, is a 33-year-old female with deafness, ataxia, and GI dysmotility. Patient 2, carrying the m.3243A>G mutation, had progressive mitochondrial disease with deafness, diabetes, stroke-like episodes, cognitive impairment, cardiac abnormality, epilepsy, and GI dysmotility. She died at the age of 36. Patient 3, with the m.3243A>G mutation, was the aunt of patient 2. She progressively developed symptoms of mitochondrial disease, including cognitive impairment, deafness, and GI dysmotility. She died at the age of 64. Patient 4 harboured the m.8344A>G mutation with progressive development of mitochondrial disorders involving myoclonus, epilepsy, cognitive impairment, and cardiac abnormalities. He died at age 56 years. The heteroplasmy levels of pre-mortem tissue homogenates from these patients are shown in the supplementary material, Table S2.
