# Supplementary material for: Inherited pathogenic mitochondrial DNA mutations and gastrointestinal stem cell populations
Source: J Pathol. 2018 Nov 5;246(4):427–32. doi: 10.1002/path.5156 (PMC6282723; doi:10.1002/path.5156)
Supplement: Supplementary file 2 — Supplementary figure legends [file PATH-246-427-s007.docx]

**Inherited pathogenic mitochondrial DNA mutations and gastrointestinal stem cell populations**

**Su T *et al.* J Pathol 2018 (DOI: 10.1002/path.5156)**

**Supplementary figure legend**

**Figure S1.** Quantitative measurement of (A) COX4 and (B) SDHA level in the gastrointestinal epithelium and smooth muscle. *Z*-scores of COX4 and SDHA for each patient case were calculated and classified according to the age-matched control population. The number of crypts for COX4 analysis was as follows: *n* (P1 SI) = 70; *n* (control) =128; *n* (P2 SI) = 28, *n* (control) = 48; *n* (P2 stomach) = 6, *n* (control) = 36; *n* (P3 colon)=20, *n* (control) = 91. The number of crypts for SDHA analysis was as follows: *n* (P1 SI) = 20; *n* (control) = 83; *n* (P2 SI)= 30, *n* (control) = 47; *n* (P2 stomach) = 7, *n* (control) = 44; *n* (P3 colon)=20, *n* (control) = 79. All areas of oesophageal epithelium and colonic smooth muscle from the section were chosen for quantification. Patient data were compared with data from two controls for the stomach, three controls for the colon, the oesophagus and the SI of patient 2, and four controls for the SI of patient 1
