# Supplementary figures and images for: Inherited pathogenic mitochondrial DNA mutations and gastrointestinal stem cell populations
Source: J Pathol. 2018 Nov 5;246(4):427–32. doi: 10.1002/path.5156 (PMC6282723; doi:10.1002/path.5156)

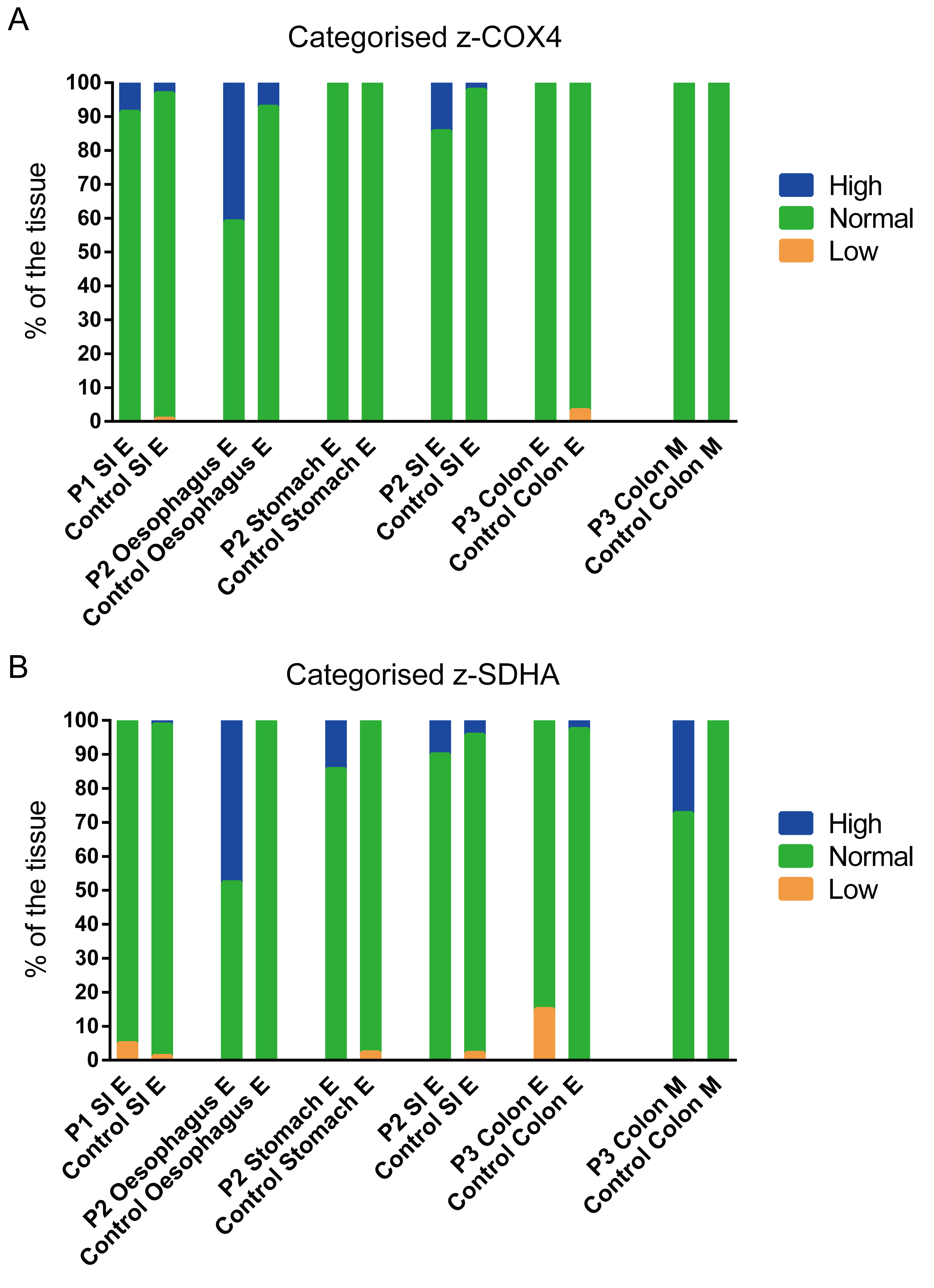

Supplement: Supplementary file 3 — Figure S1. Quantitative measurement of COX4 and SDHA level in the gastrointestinal epithelium and smooth muscle [file PATH-246-427-s005.tif]
