# Supplementary material for: Inherited pathogenic mitochondrial DNA mutations and gastrointestinal stem cell populations
Source: J Pathol. 2018 Nov 5;246(4):427–32. doi: 10.1002/path.5156 (PMC6282723; doi:10.1002/path.5156)
Supplement: Supplementary file 4 — Table S1. Information of the subjects and the obtained tissue [file PATH-246-427-s002.docx]

**Inherited pathogenic mitochondrial DNA mutations and gastrointestinal stem cell populations**

**Su T *et al.* J Pathol 2018 (DOI: 10.1002/path.5156)**

### Table S1. Information of the subjects and the obtained tissue

| Subject | Age  (years) | Gender | MtDNA genotype | Tissue | Tissue  component |
| --- | --- | --- | --- | --- | --- |
| P1 | 30 | F | m.3243A > G | Small intestine | E and M |
| P2 | 36 | F | m.3243A > G | Oesophagus, stomach, and small intestine | E and M |
| P3 | 64 | F | m.3243A > G | Colon | E and M |
| P4 | 58 | M | m.8344A > G | Colon | E and M |
| C1 | 30 | F | WT | Small intestine | E |
| C2 | 31 | F | WT | Small intestine | E |
| C3 | 31 | F | WT | Small intestine | E |
| C4 | 34 | F | WT | Small intestine | E |
| C5 | 37 | F | WT | Small intestine | E |
| C6 | 37 | F | WT | Small intestine | E |
| C7 | 59 | F | WT | Colon | E and M |
| C8 | 61 | F | WT | Colon | E and M |
| C9 | 62 | M | WT | Colon | E and M |
| C10 | 64 | F | WT | Colon | E and M |
| C11 | 64 | M | WT | Colon | E |
| C12 | 41 | M | WT | Oesophagus and stomach | E |
| C13 | 44 | M | WT | Oesophagus and stomach | E |
| C14 | 57 | F | WT | Oesophagus | E |
| C15 | 58 | F | WT | Oesophagus | E |

E, epithelium; M, muscle. The age indicates the age of the patients when the tissue was collected.
