# Supplementary material for: Inherited pathogenic mitochondrial DNA mutations and gastrointestinal stem cell populations
Source: J Pathol. 2018 Nov 5;246(4):427–32. doi: 10.1002/path.5156 (PMC6282723; doi:10.1002/path.5156)
Supplement: Supplementary file 5 — Table S2. Heteroplasmic levels of pathogenic mtDNA mutations measured in various tissues of the four patients [file PATH-246-427-s004.docx]

**Inherited pathogenic mitochondrial DNA mutations and gastrointestinal stem cell populations**

**Su T *et al.* J Pathol 2018 (DOI: 10.1002/path.5156)**

### Table S2. Heteroplasmic levels of pathogenic mtDNA mutations measured in various tissues of the four patients

| **Type** | **Tissue** | **Mutation load (%)** | | | |
| --- | --- | --- | --- | --- | --- |
|  |  | **P1**  **(m.3243A>G)** | **P2**  **(m.3243A>G)** | **P3**  **(m.3243A>G)** | **P4**  **(m.8344A>G)** |
| Entirely or partially mitotic | Blood | 27 | 21 | 5 |  |
|  | Urine | 38 | 33 | 72 | 70 |
|  | Hair shafts |  | 51 |  |  |
| Post-mitotic | Skeletal muscle | 63 | 64 (PM) | 72 (PM) | 91 (PM) |
|  | Cardiac muscle |  | 77 (PM) | 85 (PM) | 83 (PM) |

PM, post-mortem.
