# Supplementary material for: Inherited pathogenic mitochondrial DNA mutations and gastrointestinal stem cell populations
Source: J Pathol. 2018 Nov 5;246(4):427–32. doi: 10.1002/path.5156 (PMC6282723; doi:10.1002/path.5156)
Supplement: Supplementary file 6 — Table S3. Primer sequences used for pyrosequencing to quantify m.3243A>G and m.8344A>G mutation levels [file PATH-246-427-s003.docx]

**Inherited pathogenic mitochondrial DNA mutations and gastrointestinal stem cell populations**

**Su T *et al.* J Pathol 2018 (DOI: 10.1002/path.5156)**

**Table S3.** Primer sequences used in the pyrosequencing assay to quantify m.3243A>G and m.8344A>G mutation levels

| **m.3243A>G primer design** | **m.8344A>G primer design** |
| --- | --- |
| Genbank Accession number: [NC_012920](http://www.ncbi.nlm.nih.gov/entrez/query.fcgi?db=Nucleotide&cmd=Search&term=NC_012920) | |
| 5′-Biotin forward primer (nt3143–3163) | 5′-Biotin forward primer (nt 8240–8264) |
| Reverse primer (nt 3331–3353) | Reverse primer (nt8363–8387) |
| Sequencing primer (nt 3244–3258) | Sequencing primer (nt 8347–8367) |
