# Supplementary material for: Inherited pathogenic mitochondrial DNA mutations and gastrointestinal stem cell populations
Source: J Pathol. 2018 Nov 5;246(4):427–32. doi: 10.1002/path.5156 (PMC6282723; doi:10.1002/path.5156)
Supplement: Supplementary file 7 — Table S4. Antibodies and concentrations used in the immunofluorescence assay [file PATH-246-427-s006.docx]

**Inherited pathogenic mitochondrial DNA mutations and gastrointestinal stem cell populations**

**Su T *et al.* J Pathol 2018 (DOI: 10.1002/path.5156)**

**Table S4.** Antibodies and concentrations used in the immunofluorescence assay

| **Primary antibody** | | **Secondary antibody** | | **Tertiary antibody** | |
| --- | --- | --- | --- | --- | --- |
| **Name** | **Conc.**  **(µg/ml)** | **Name** | **Conc.**  **(µg/ml)** | **Name** | **Conc.**  **(µg/ml)** |
| Mouse anti-NDUFB8 (Abcam, ab110242) | 20 | Biotinylated goat anti-IgG1 (Invitrogen, A10519) | 10 | Streptavidin-conjugated Alexa Fluor 488 (Thermo Fisher Scientific, S32354) | 10 |
| Mouse anti-COX4 (Abcam, ab110261) | 5 | Goat anti-mouse IgG2a Alexa Fluor 546 (Thermo Fisher Scientific, A21133) | 10 | N/A | |
| Mouse anti-SDHA (Abcam, ab14715) | 5 | Goat anti-mouse IgG1 Alexa Fluor 647 (Invitrogen, A21240) | 10 | N/A | |
